# Deep learning models for the early detection of maize streak virus and maize lethal necrosis diseases in Tanzania

**DOI:** 10.3389/frai.2024.1384709

**Published:** 2024-08-16

**Authors:** Flavia Mayo, Ciira Maina, Mvurya Mgala, Neema Mduma

**Affiliations:** ^1^Computational and Communication Science Engineering (CoCSE), The Nelson Mandela African Institution of Science and Technology (NM-AIST), Arusha, Tanzania; ^2^Electrical and Electronic Engineering, Dedan Kimathi University of Technology, Nyeri, Kenya; ^3^Institute of Computing and Informatics, Technical University of Mombasa, Mombasa, Kenya

**Keywords:** deep learning models, maize diseases, early detection, convolutional neural network, vision transformer, maize streak virus, maize lethal necrosis

## Abstract

Agriculture is considered the backbone of Tanzania’s economy, with more than 60% of the residents depending on it for survival. Maize is the country’s dominant and primary food crop, accounting for 45% of all farmland production. However, its productivity is challenged by the limitation to detect maize diseases early enough. Maize streak virus (MSV) and maize lethal necrosis virus (MLN) are common diseases often detected too late by farmers. This has led to the need to develop a method for the early detection of these diseases so that they can be treated on time. This study investigated the potential of developing deep-learning models for the early detection of maize diseases in Tanzania. The regions where data was collected are Arusha, Kilimanjaro, and Manyara. Data was collected through observation by a plant. The study proposed convolutional neural network (CNN) and vision transformer (ViT) models. Four classes of imagery data were used to train both models: MLN, Healthy, MSV, and WRONG. The results revealed that the ViT model surpassed the CNN model, with 93.1 and 90.96% accuracies, respectively. Further studies should focus on mobile app development and deployment of the model with greater precision for early detection of the diseases mentioned above in real life.

## 1 Introduction

Tanzania’s economy is predominantly centered around agriculture, and the country gains from a wide range of agricultural activities, such as livestock, essential food crops, and many cash crops (Oxfordbusinessgroup, 2018). In Tanzania, agricultural output accounts for about 29.1% of the country’s Gross Domestic Product (GDP). It also employs 67% of the labor force, a paramount supplier of food, raw materials for industry, and foreign exchange (International Trade Administration, 2021). Moreover, as agronomy production is far too low, food demand is increasing dramatically (Dewbre et al., 2014). Farmers, scientists, researchers, analysts, specialists, and the government are working hard to enhance agricultural production to meet growing needs (Panigrahi et al., 2020). However, crop diseases continue to be a challenge affecting major food security crops like maize (Savary and Willocquet, 2020). Maize is a very crucial and important crop in Tanzania, contributing significantly to the country’s agricultural sector (Maiga, 2024). However, maize leaf diseases such as Maize Streak Virus and Maize Lethal Necrosis, pose a severe threat to maize production with the potential to reduce yield (Shepherd et al., 2010; Mahuku et al., 2015; Kiruwa et al., 2020). Early detection of these diseases is crucial for implementing timely preventive measures and mitigating yield losses (Boddupalli et al., 2020; Haque et al., 2022). Traditional visual analysis methods for disease detection in crops are prone to errors, labor-intensive, and time-consuming. Moreover, these methods have been observed to identify diseases at a later stage, potentially leading to more harm to the crops (Toseef and Khan, 2018; Gong and Zhang, 2023). These traditional methods rely heavily on the expertise of farmers, plant pathologists, and agriculture experts. Additionally, the subjective nature of these methods can lead to inconsistent diagnoses among different experts.

**Figure 1:**
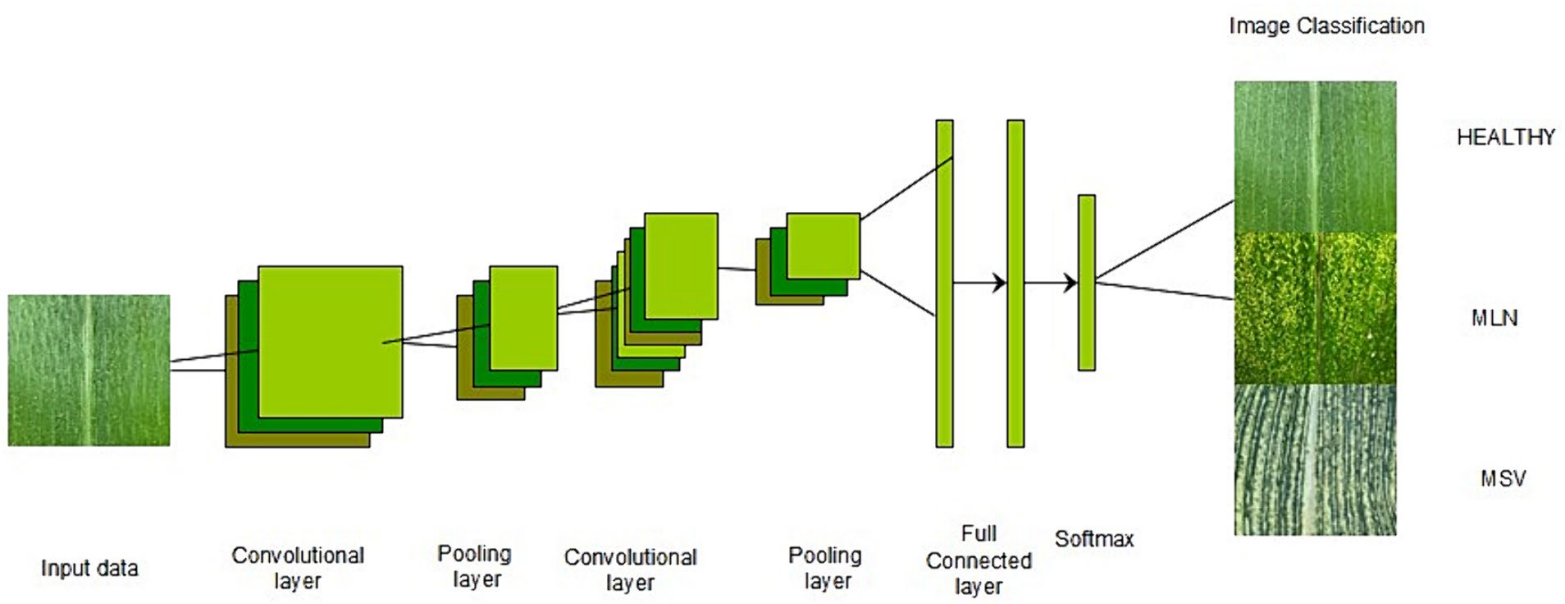
CNN architecture (Voulodimos et al., 2018).

Recently, technology has been used to improve yields in agriculture, whereby researchers have devised several solutions, including image processing and object detection using deep learning models (Panigrahi et al., 2020). Deep learning (DL) is a branch of machine learning that involves training artificial neural networks to learn from large volumes of data and make predictions. Moreover, it is known for its ability to use many processing layers to discover patterns and structures in large datasets (Rusk, 2015). It moreover automatically extracts features from the data, making them suitable for various applications, such as image recognition, natural language processing, speech recognition, and autonomous systems (Ho, 2016). It has become widely known for its potential and advanced ability to efficiently process large numbers of images, yielding reliable outcomes. It is doing very well in many fields, including agriculture (Kamilaris and Prenafeta-Boldú, 2018). During the last few years, many crops have become accustomed to detecting, classifying, and assessing a broad spectrum of diseases, pests, and stresses (Singh et al., 2016; Panigrahi et al., 2020; Haque et al., 2022). For the past several years, deep learning achievements in computer vision tasks have strongly depended on Convolutional Neural Networks (CNNs) (Raghu et al., 2021). CNNs prevail in the domain of computer vision as a foundation for various applications, such as image classification (Sibiya and Sumbwanyambe, 2019; Darwish et al., 2020; Syarief and Setiawan, 2020; Atila et al., 2021; Chen et al., 2021; Liu and Wang, 2021; Haque et al., 2022), object detection (Zhang et al., 2020; Liu and Wang, 2021; Maxwell et al., 2021; Roy et al., 2022) and image segmentation (Gayatri et al., 2021; Liu and Wang, 2021; Loyani and Machuve, 2021; Maxwell et al., 2021; Sibiya and Sumbwanyambe, 2021). The CNN architecture consists of components such as a convolutional layer, a pooling layer, a fully connected layer, and activation functions (Bharali et al., 2019; Francis and Deisy, 2019; Jasim and Al-Tuwaijari, 2020), as shown in Figure 1.

**Figure 2:**
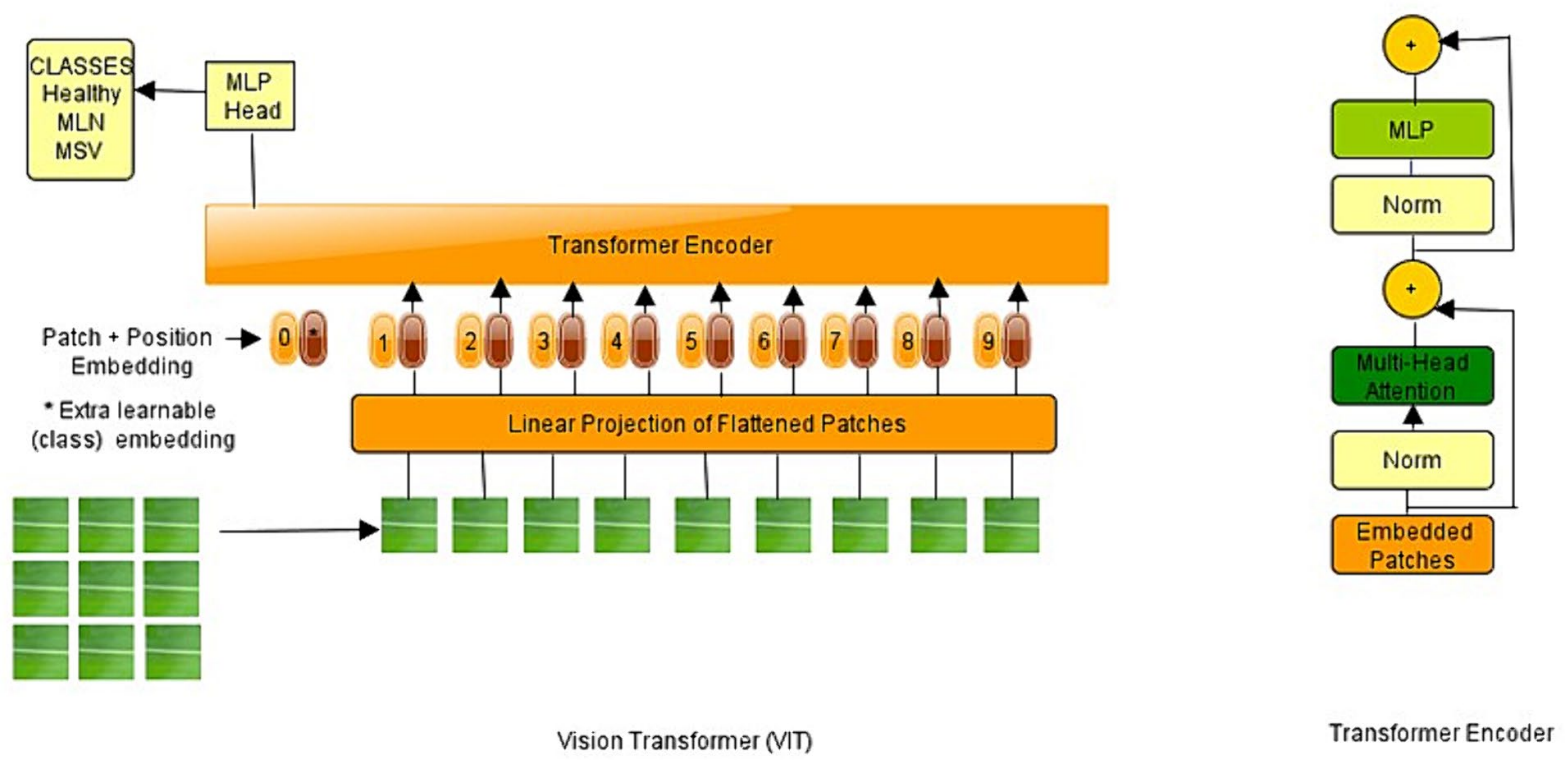
Vision transformer architecture (Dosovitskiy et al., 2020).

Natural language processing has been performed using transformer architecture, and vision transformers have produced outstanding outcomes compared to CNNs (Vaswani et al., 2017; Qi et al., 2022). Researchers have recently adapted transformers to computer vision applications, inspired by the significant success of transformer architectures in the field of NLP. The Vision Transformer (ViT) has achieved cutting-edge performance on various image recognition benchmarks. In addition to image classification, transformers have been used to solve a variety of computer vision problems, including object identification, semantic segmentation, image processing, and video interpretation. Because of their superior performance, an increasing number of academics are proposing transformer-based models for improving a wide range of visual tasks (Han et al., 2023). ViT works by implementing a transformer-like architecture over image patches. Images are divided into fixed-size patches, which are then linearly embedded. Position embeddings are then added, then the resulting vector sequence is fed into a standard transformer encoder. The standard approach of adding an extra learnable classification token to the sequence is used to perform classification (Vaswani et al., 2017; Dosovitskiy et al., 2020). The sequence of the 1D array is passed to the transformer structure. To process 2D image patches, the 2D patches are extracted from the first, and then they are reshaped to create 1D arrays that are suitable for the ViT structure. They are added to the positional encoder to finish preparing the patch embedding for the next layer. The positional encoder aids the network in remembering the relative position of the patches with one another. Inputs are then normalized with the normalization layer before entering the transformer block. The multi-head attention layer is the most important aspect of this block. The multi-head attention layer calculates weights to assign higher values to the more important areas. In other words, network attention is focused on the most important parts of the network. The output of the multi-head attention layer is a linear combination of each head (Borhani et al., 2022). Figure 2 shows the ViT architecture inspired by Vaswani et al. (2017).

Both the ViT and CNN models have achieved state-of-the-art results in various computer vision tasks, including plant disease detection. However, the relative performance of the model would depend on the specific dataset, model architecture, and training hyperparameters used in a certain study. A lot of various techniques have been developed and proposed for the detection of diseases in general. The most adopted techniques CNN and ViT have shown great performance when used separately Therefore, this study aimed to develop combined deep-learning models for the early detection of Maize Streak Virus (MSV) and Maize Lethal Necrosis (MLN) diseases in maize plants based on images obtained and collected directly from the field, allowing the model to be trained with real data. The grand purpose is to utilize the maize imagery datasets collected from farms and made available in open source to the research community for future studies on MLN and MSV infections, by introducing, an approach that enhances the effectiveness and efficiency of these diseases in maize. Hence this paper fills a gap existing in a debate between the most quality and reliable model for detection of maize diseases.

## 2 Related works

The diagnosis of a wide variety of plant diseases and pests has shown encouraging and remarkable results when employing deep learning techniques in computer vision, such as CNNs. A convolutional neural network deep learning model was developed to analyze images of healthy and unhealthy plant leaves. A total of 87,848 images in an open database with 25 distinct plants in 58 distinct categories of healthy and unhealthy images were trained using five model architectures, AlexNet, AlexNetOWTBn, GoogLeNet, Overfeat, and VGG. VGG was the most common architecture for detecting plant diseases, with a higher success rate. Implementation was performed using the Torch71 machine learning computational framework, which uses the LuaJIT programming language. The model’s exceptionally excellent performance makes it suitable as a vital early warning or advising tool (Ferentinos, 2018). This study was conducted in Athens, Greece, to detect many plant diseases and not specifically for the detection of maize streak virus and lethal maize necrosis.

Another deep-learning model was developed to detect maize diseases in Indonesia. The study used a classification approach to detect 3 diseases, Cercospora, northern leaf blight, and common rust. A support vector machine, k-nearest neighbor, and decision tree were used to classify the maize leaf images, and seven other CNN architectures were used to analyze the maize leaf images. The architectures used included ResNet50, GoogleNet, VGG19, AlexNet, Inception-V3, VGG16, ResNet110 and VGG19. The data consisted of 200 images that were divided into 4 classes, 50 images per class with a size of 256×256 pixels. However, AlexNet and SVM were the best methods for feature extraction and image classification of maize leaf diseases. This study used fewer samples (200 images), which were collected in Asia (Syarief and Setiawan, 2020).

Additionally, a Mobile-DANet model was developed to identify 8 maize crop diseases, gibberella ear rot, maize eyespot, crazy top, gray leaf spot, Goss’s bacterial wilt, common smut, phaeosphaeria spot, and southern rust. Except for some samples, the results of the Mobile-DANet model demonstrated that the majority of the images and maize diseases were correctly identified. Mobile-DANet correctly detected samples with phaeosphaeria spots with a probability of 0.71. Similarly, the model accurately detected gibberella ear rot and southern rust disease, with probabilities of 0.83 and 0.93, respectively. China served as the study location, and this study focused on maize images other than MSV and MLN images. The model employed in the study is Mobile-DANet (Chen et al., 2021).

Furthermore, another study from India proposed a deep convolutional neural network to detect healthy and diseased images of maize leaves. The dataset contained 5,939 images of maize leaves. The dataset consisted of images of three diseases, Maydis leaf blight (MLB), Sheath blight (BLSB), Turcicum leaf blight (TLB), and banded leaf, as well as healthy maize leaves. The study employed the Inception-v3 network structure, as well as three more different models were developed using the normal training procedure (Haque et al., 2022).

In Cairo, Egypt, a classification model for the identification of common rust, northern leaf blight, healthy maize leaves, and gray leaf spots was developed. To identify plant diseases, an ensemble model composed of two pre-trained convolutional neural networks, VGG19 and VGG16, was used to distinguish between the leaves in healthy photos and the leaves in unhealthy photos. The outcomes show how well the suggested strategy works, outperforming alternative methods for VGG19. Even though the created model performed well, this study struggled with the categorization of unbalanced data, and the dataset employed lacked sufficient images to properly train CNNs that were created from scratch (Darwish et al., 2020).

A model for the recognition of common rust (Puccinia sorghi), gray leaf spot (Cercospora), and northern corn leaf blight (Exserohilum) from healthy leaves was developed due to the impacts of these diseases on the majority of the maize plantations in South Africa. Neuroph was used for training the convolution neural network to recognize and classify images of maize. CNN was quite correct in identifying these diseases. This research was restricted to the neuroph framework of the Java neural network, which is an integrated environment for developing and deploying neural networks to Java programs, despite the model’s strong performance (Sibiya and Sumbwanyambe, 2019).

A similar study was conducted by Sibiya and Sumbwanyambe (2021) to develop a CNN deep learning model. The diseased leaf area was calculated using segmentation by the threshold on diseased images of leaves of maize impacted by common rust disease. This information was used to create ambiguous decision guidelines in assigning common rust images to severity groups with images created using this proposed approach. The VGG-16 network, trained with images generated using this suggested method, achieved a higher testing and validation accuracy when tested on photos of common rust illness in 4 stages of severity (early stage, middle stage, late stage, and healthy stage). Despite the good performance of the developed model, this study was limited to only the image segmentation approach, which tends to partition a digital image into multiple segments. Furthermore, the study used a CNN architecture, which lacked a detailed description.

Arnaud et al. (2022) from Kenya developed a deep learning model to examine, in contrast, 6 convolutional neural network architectures. Transfer learning was employed for model training, and the architectures used included EfficientNet b7, VGG19, SqueezeNet, GoogleNet, AlexNet, and DenseNet. The study analyzed four hyperparameters: the batch size, learning rate, number of epochs, and number of optimizers. An open-source dataset with 4,082 photos was used. DenseNet121 outperformed other models by achieving a higher accuracy and F1 score. DenseNet121 was trained with batch 32, a learning rate of 0.01, and stochastic gradient descent (SGD) as the optimizer. In general, various techniques for detecting plant diseases have been proposed. These techniques have shown good performance; however, no studies have focused on building a combined deep-learning model for the detection of MSV and MLN together, and there is no publicly available dataset containing images of maize leaves infected by MSV and MLN. Moreover, several studies have used a large number of images from online sources, which might not accurately represent field scenarios. As a result, this study aimed to develop a combined deep learning model for MSV and MLN detection based on images collected directly from the field, allowing the model to be trained with real data. The dataset will be made available in open source to the research community for future studies on MLN and MSV infections. Furthermore, the majority of the studies employed transfer learning methods, and the scope of their studies was not in Tanzania.

## 3 Materials and methods

### 3.1 Overview of the proposed method

**Figure 3:**
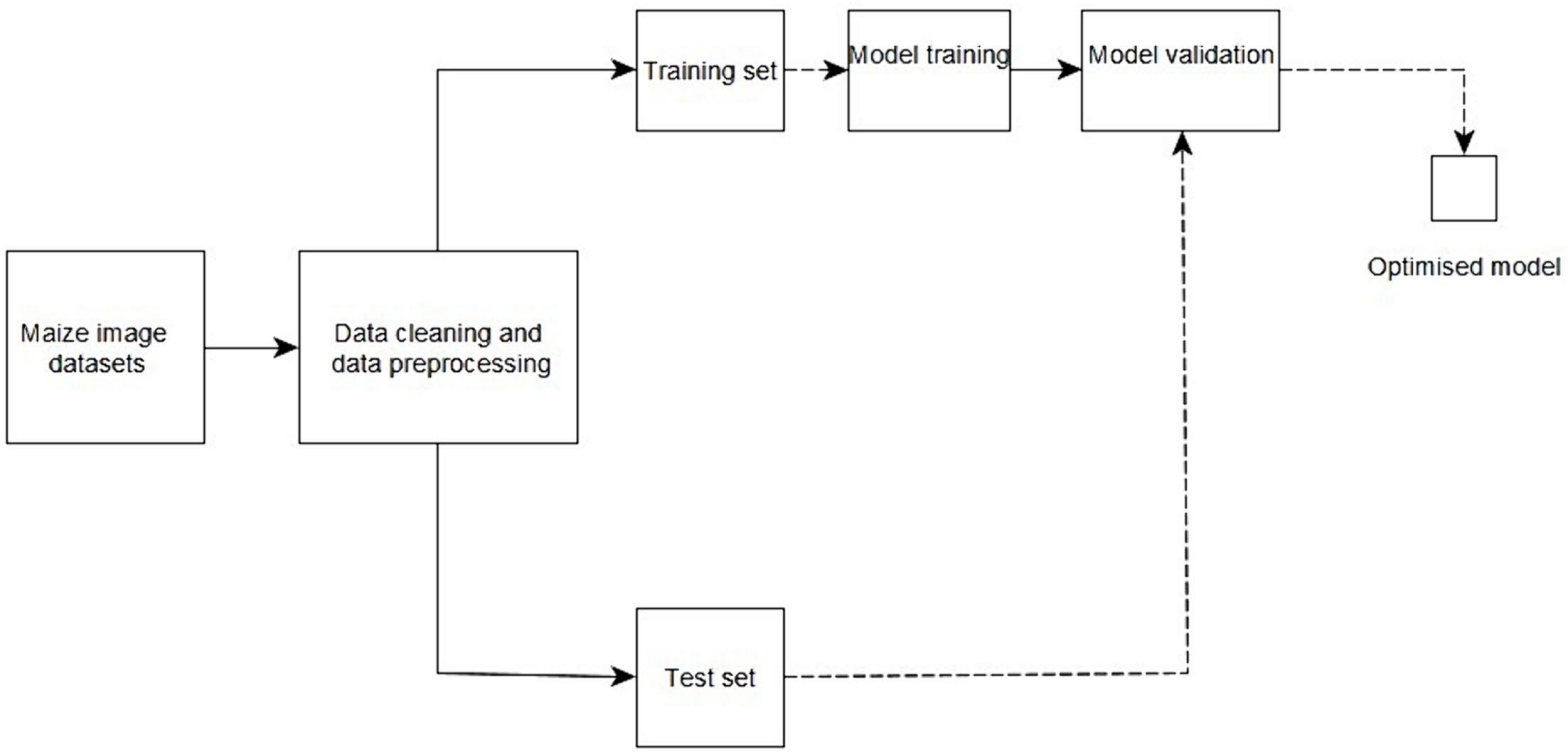
Block diagram summary for the proposed work.

Figure 3 provides an overview of the proposed method from the acquisition of data to model development, model validation, and delivery of an optimized model. Images of healthy and diseased maize leaves were collected from the farms. The image datasets were then pre-processed and divided into training and testing sets. The models were then trained and tested to evaluate the performance and accuracy of the created models.

### 3.2 The dataset

**Figure 4:**
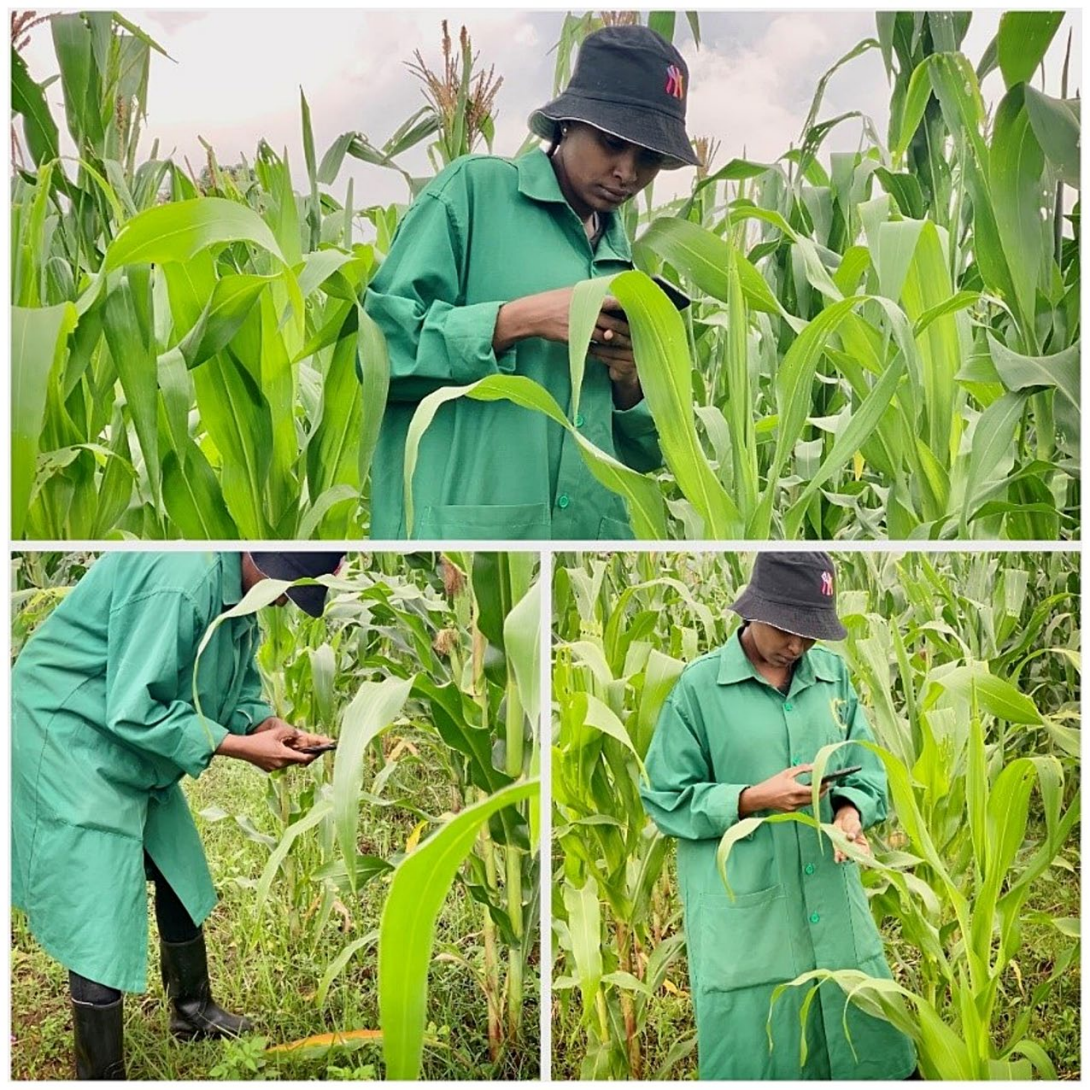
Researcher collecting imagery leaves in maize farms.

**Figure 5:**
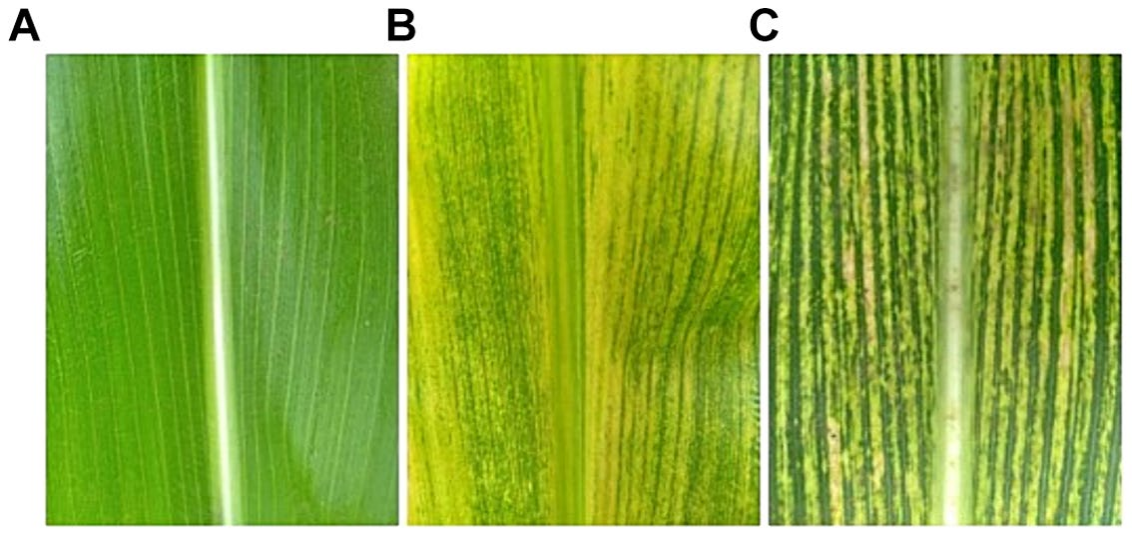
Examples of imagery data from maize dataset where **(A)** Healthy, **(B)** MLN, **(C)** MSV.

The datasets were collected from three regions which are Arusha, Kilimanjaro, and Manyara. These regions were selected due to having a large number of farmers across the country. The focus of the dataset collection was on the affected maize plants. Two main diseases MSV and MLN were observed from the leaves and images were captured. Leaves were selected from the middle tier of the maize plants. This tier was chosen to provide a consistent basis for comparison, as leaves at different heights may exhibit varying levels of disease symptoms. They were collected during the mid-season phase of the growing season. This phase was selected because it is when the symptoms of Maize Streak Virus (MSV) and Maize Lethal Necrosis (MLN) are most prominent and easily identifiable. Moreover, the study focused on two widely cultivated maize varieties in Tanzania: Situka M1 and T105. These varieties were chosen due to their regional prevalence and known susceptibility to MSV and MLN. By including two varieties, the study aimed to ensure that the model is robust and generalizable across different genetic backgrounds. The process of data collection took a period of (6) months, starting from February to July, the process involved plant pathologists to be able to identify the symptoms of the diseases. The Open Data Kit (ODK) tool installed in a smartphone was used to capture these images. All the images were captured in the format of a Joint Photographic Group (JPG). At the end of data collection, 27,660 images were obtained which were sufficient for model development. The distribution of these images was 9,145 healthy images, 8,604 MLN images, and 9,911 MSV images. To prepare the proposed model to be able to identify images other than maize leaf images, 675 more images of different things were acquired from open-access databases to be included for training the model. Figure 4 shows the researcher collecting data in the field, and Figure 5 shows the sample image data samples captured from the three classes that were collected from the field. Image labeled (a) is an image of a maize leaf that is healthy, image labeled (b) is an image of a maize leaf affected with Maize Lethal Necrosis (MLN) and the last image labeled (c) is an image of a maize leaf affected by Maize Streak Virus (MSV).

### 3.3 Data cleaning and preprocessing

This is a very crucial stage, where all the collected data is cleaned and ensured it is free of any erroneous or fraudulent information. This process normally uses various tools and software (Lee et al., 2021). In the data-cleaning stage, the following steps were conducted.

#### 3.3.1 Removing duplicates and cropping

In this step, duplicate images from the three classes, Healthy, MSV, and MLN, were removed using the VisiPics tool (Arora et al., 2016). The tool was selected because of easy usage and it functions very well in eliminating exactly similar images. In total there were 27,660 images collected from the field before removing duplicates, 747 images were found duplicates and deleted. 26,913 images remained after removing duplicates. Table 1 lists the total number of images from the three classes, before and after the duplicates have been removed. The images were also cropped manually to remove unnecessary background so that maize leaf would be the main focus. This is seen in Figure 5.

**Table 1:** Number of images before and after duplicate images.

Classes	Numbers of images before duplicates	Duplicate images	Number of images after duplicates
Healthy	9,145	530	8,615
MLN	8,604	26	8,578
MSV	9,911	191	9,720
Total	27,660	748	26,913

#### 3.3.2 Labeling and resizing

The labeling process was conducted with the help of a tool named bulk rename utility to fasten the labeling process. Image labeling was done by naming the data to the corresponding classes. These images were ensured to have a jpg format to be able to function during the development of the model. The labeling involves a process for determining what number of images will be used for model training and model validation. The image dataset was also resized according to the proposed deep-learning model requirements. Images employed to train and test the CNN model were resized to a uniform pixel of size 256*256, and images used to train and test the Vit model were resized to a uniform pixel of size 200*200. Proposed models.

This study focused on developing two deep learning models, a Convolutional Neural Network (CNN) and a Vision Transformer (ViT), for the early detection of Maize Streak Virus (MSV) and Maize Lethal Necrosis (MLN) diseases.

### 3.4 Model development

#### 3.4.1 CNN

CNNs are a class of deep learning algorithms primarily used for image recognition and classification. They are designed to recognize local patterns in the input image. This algorithm comprises key components that include convolution layers, pooling layers, fully connected layers, and activation functions. Convolutional layers are used to apply convolution operations to the input image, passing the results to the next layer. Pooling layers play the role of down-sampling operations to reduce the dimensionality of the feature maps, which assists in the reduction of overfitting and computational complexity. Fully connected layers are commonly used at the end of the network to output a class score, however just like traditional neural networks they connect every neuron in one layer to every neuron in the next layer. Activation functions are used to introduce non-linearity to the model.

CNN model was developed with a total of 27,588 images from four classes (Healthy, MLN, MSV, and WRONG). The dataset was split into 80% for the training set and 20% for the testing set for all four classes. Because of the large number of images, the model was trained in four groups of batches where the output weights that were utilized in training the first batch were employed as input in training the second batch, then the same thing for the third and fourth batch. The first three batches each contained 6,000 datasets. The datasets were split into 4,800 images for the training set and 1,200 images for the test set for each batch in (Healthy, MSV, and MLN); however, for the WRONG class in the training set, 540 images were included, and for the test set, 135 images were included, maintaining an 80:20 ratio for each class. For the fourth batch, the model was trained using the remaining 8,913 datasets. The dataset was again split into an 80:20 ratio for the training set and the test set, resulting in 7,131 samples for training and 1,782 samples for testing for Healthy, MSV, and MLN, where the number of the WRONG image class remained the same. A sequential model was employed in this implementation that defined 5 convolutional layers, and each layer was followed by a max pooling layer. The first convolution layer had 16 filters; the second convolution layer had 32 filters; and the third to fifth layers had 64 filters. These were then followed by a flattening layer and a dense layer with 512 neurons. A rectified linear unit (ReLU) was employed as an activation function in all the convolutional layers. The number of classes was represented by the output dense layer, which had 4 neurons with a softmax activation function. The images were rescaled by (1.0/255) and resized to 256 × 256 pixels. The hyperparameters used for training the CNN model and their values are shown in Table 2.

**Table 2:** Hyperparameters used for training the CNN model.

Parameter	Value
Epoch	50
Batch size	32
Steps per epoch	167
Optimizers	Adam
Losses	Categorical_crossentropy
Metrics	Accuracy, Precision, Recall, F-measure

#### 3.4.2 ViT

Vision Transformers (ViT) represents a novel approach to image recognition tasks by utilizing the transformer architecture which was initially created for challenges related to natural language. Important ViT components include patch embedding, transformer encoder, self-attention mechanism, and position embedding. In patch embedding an input image is split into fixed-size patches, and each patch is linearly embedded into a vector. These embeddings are then combined to form a sequence. The sequence of patch embeddings is processed through multiple layers of the transformer encoder. Each encoder layer consists of a multi-head self-attention mechanism and feed-forward neural networks. The self-attention mechanism is what allows the model to weigh the importance of different patches in the image enabling it to capture long-range dependencies and contextual information. Since transformers do not have a built-in notion of spatial relationships, position embeddings are added to the patch embeddings to retain the spatial information of the image.

The ViT model was developed with a dataset consisting of a total of 6,675 samples from four classes (HEALTHY, MLN, MSV, and WRONG). The images were resized to a uniform size of 200×200 pixels. The ViT model architecture comprises patch embedding, positional embedding, 12 transformer layers, and a classification head. Each transformer layer includes 12 attention heads in the multi-head attention mechanism, and the feedforward neural networks in the transformer have a dimensionality of 3,072. Each patch in the image has a size of 25, and the number of output classes is 3, corresponding to the number of classes in the dataset. The hidden dimensionality of the transformer model is 768, and a dropout rate of 0.1 was applied. The activation function used in this model was the Gaussian error linear unit (GELU). The hyperparameters used for training the ViT model are shown in Table 3.

**Table 3:** Hyperparameters used for training the ViT model.

Parameters	Value
Epoch	50
Steps per epoch	154
Batch size	32
Optimizer	Adam
Metric	Accuracy
Learning rate	0.0001
Losses	categorical-Crossentropy

### 3.5 Experimental setup

The experiment for this study was conducted on a machine running Windows 10 with an Intel(R) Core (TM) i5-4200U CPU @ 1.60 GHz and 2.30 GHz with an installed RAM of 8 GB and a 64-bit operating system. Both the CNN and ViT models were trained online using Google Collab, which consists of Python3 as the run-time and a GPU as the hardware accelerator. The implementation was carried out using the Keras library with TensorFlow on the backend. The language used during model training was Python because of its ability to provide a variety of freely available machine-learning libraries.

## 4 Results and discussion

### 4.1 CNN model training results

**Figure 6:**
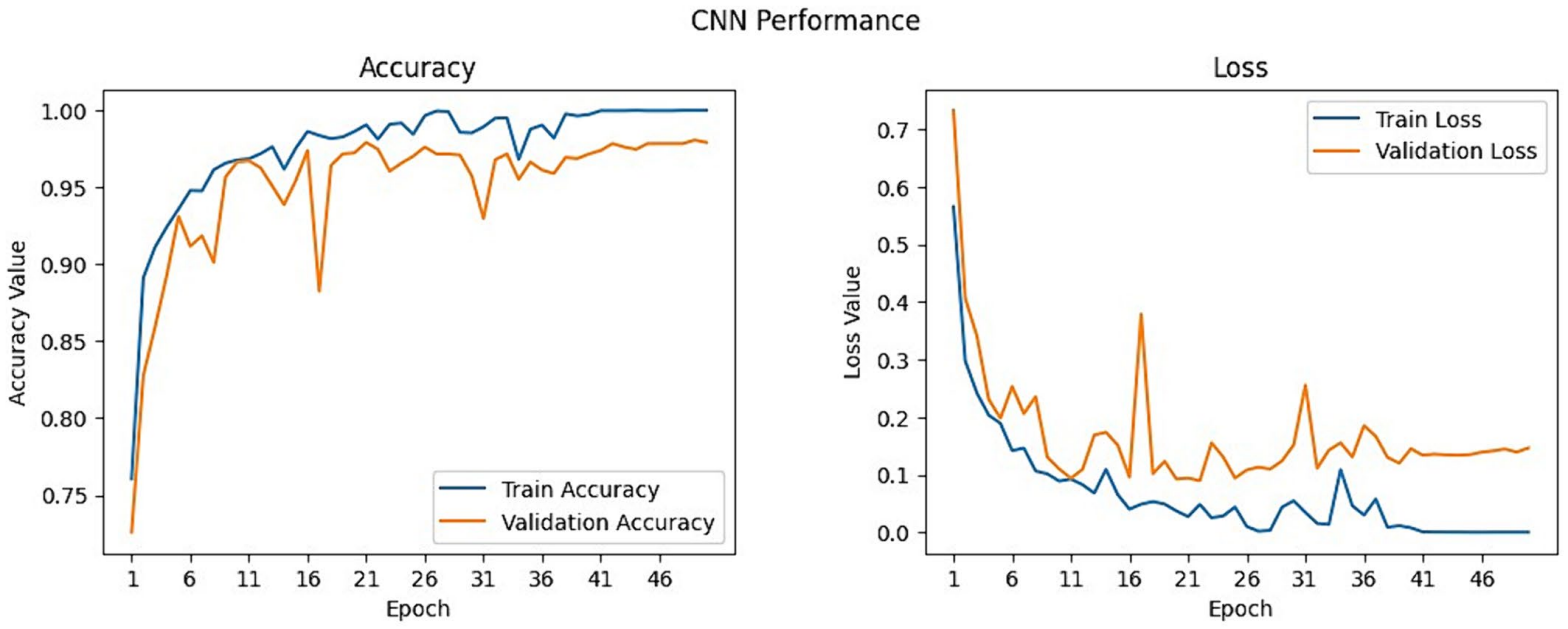
Training and validation plot for CNN model.

The model training results show that the second batch got the highest validation accuracy of 0.9791 and a low validation loss of 0.1465. The average of the validation accuracy for the entire training for all datasets from all 4 batches is 0.90965. The results for model performance recorded during the 1st to the 50th epoch for each of the four batches are summarized in Table 4. Figure 6 on the left shows the CNN training accuracy and loss curve of over 50 epochs. The results for accuracy over the epoch graph show that the validation accuracy increased rapidly up to the 5th epoch, then remained steady at around 90% exhibiting fluctuations up to the 16th epoch where it dropped to 0.8824 on the 17th epoch and went high again remaining steady in the 0.90 with fluctuations up to the last epoch and reaching a peak of 0.9790. Meanwhile, the training accuracy increased rapidly up to the 12th epoch and followed a similar trend of remaining steady at 0.90 with fluctuations hitting a maximum accuracy of 0.9998 surpassing the validation accuracy without any significant fluctuations. This indicates that the model exhibited effective generalization. On the loss over epoch graph in Figure 6 on the right, the results demonstrate that the training loss decreases rapidly from the 1st epoch to the 10th epoch, after which it starts to fluctuate slightly, exhibiting periodic increases and decreases until the end. Meanwhile, the validation loss shows a rapid decrease from the outset until the 5th epoch, followed by a pattern of fluctuation with periodic increases and decreases until the final epoch. This shows that the model aligns closely with the characteristics of the dataset throughout both the initial and final phases of the training process.

**Table 4:** CNN model performance results.

Batches	Validation accuracy	Validation loss	Precision	Recall	F measure
Batch 1	0.9581	0.3436	1.0000	1.0000	1.0
Batch 2	0.9790	0.1465	0.9998	0.9998	0.9998
Batch 3	0.8135	1.9335	0.9882	0.9872	0.9880
Batch 4	0.8878	0.5497	0.9672	0.9625	0.9648

### 4.2 ViT model training results

**Figure 7:**
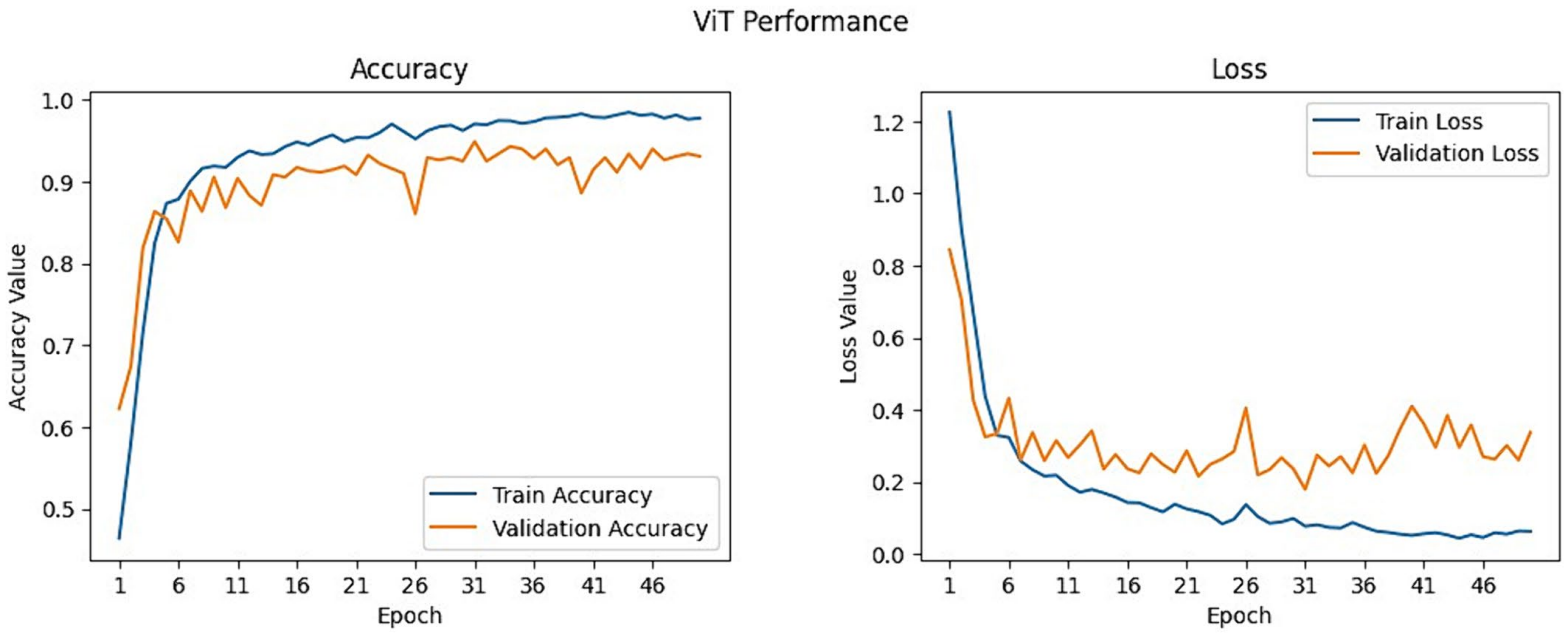
Training and validation plots for ViT model.

The ViT model was trained in only one batch. The model achieved a validation accuracy of 0.9310 and a validation loss of 0.3371. The results for model performance recorded during the 1st to the 50th epochs are plotted in Figure 7. The results for accuracy over the epoch graph show that the validation accuracy increased rapidly up to the 4th epoch, then remained steady at around 80%, and then 90% exhibiting fluctuations up to the 26th epoch where it dropped to 0.8606 on the 27th epoch, and went high again remaining steady in the 90% with fluctuations but dropped again in 40th epoch and went up to the last epoch and reaching a peak of 0.9310. Meanwhile, the training accuracy increased rapidly up to the 10th epoch and followed a similar trend of remaining steady at 90% with fluctuations hitting a maximum accuracy of 0.9777 surpassing the validation accuracy without any significant fluctuations. This indicates that the model exhibited effective generalization. On the loss over epoch graph in Figure 7 on the right, the results demonstrate that the training loss decreases rapidly from the 1st epoch to the 5th epoch, after which it starts to fluctuate slightly, exhibiting periodic increases and decreases until the end. Meanwhile, the validation loss shows a drop-down from the outset to the 4th epoch, followed by a pattern of fluctuation with periodic increases and decreases until the final epoch. This observation suggests that the model aligns closely with the characteristics of the dataset throughout both the initial and final phases of the training process.

### 4.3 Comparative analysis of accuracy results from related works

The model efficiency results from other related studies were reviewed and compared to those obtained in this work. The findings of this study fairly correlate with those from other studies (Table 5).

**Table 5:** Comparison of accuracy results from related works.

Crop diseases	Model architectures	Study reference	Highest Accuracy (%)
Variety crop diseases	AlexNet, AlexNetOWTBn, GoogLeNet, Overfeat, VGG	Ferentinos (2018)	99.53%
Cercospora, common rust, and northern leaf blight	AlexNet, virtual geometry group (VGG) 16, VGG19, GoogleNet, Inception-V3, residual network 50 (ResNet50) and ResNet101	Syarief and Setiawan (2020)	93.5%
Phaeosphaeria leaf spot, gibberella ear rot, crazy top, grey leaf spot, common smut, southern rust, Goss’s bacterial wilt, maize eyespot	Mobile-DANet	Chen et al. (2021)	95.86%
Maydis Leaf Blight, Turcicum Leaf Blight and Banded Leaf and Sheath Blight	VGG-16, VGG-19, Inception-v3, ResNet-50-v2, ResNet-101-v2, ResNet-152-v2 and InceptionResNet-v2	Haque et al. (2022)	95.99%
Variety crop diseases	VGG16 and VGG19	Darwish et al. (2020)	96.7%
Northern corn leaf blight (*Exserohilum*), common rust (*Puccinia sorghi*) and gray leaf spot (*Cercospora*)	CNN	Sibiya and Sumbwanyambe (2019)	92.85%
Maize common rust disease (Early stage, Middle stage, Late Stage, and Healthy stage.)	VGG-16	Sibiya and Sumbwanyambe (2021)	95.63%
Potato late blight and early blight are common	EfficientNet b7, VGG19, SqueezeNet, GoogleNet, AlexNet, and DenseNet	Arnaud et al. (2022)	98.34%
Maize Streak Virus and Maize lethal Necrosis	CNN and ViT	Proposed method	93.1%

## 5 Discussion

This study developed two deep learning models, CNN and ViT. Both models performed well in detecting MSV and MLN diseases in maize plants. The ViT model achieved a validation accuracy of 93.1%, whereas the CNN model achieved an overall average validation accuracy of 90.97%. These results suggest that both models are capable of detecting the presence of diseases in maize plants. Furthermore, these results are considered to be among the best examples of a good model, as a good model is expected to have an accuracy greater than 70% (Maxwell et al., 2021). However, deep learning models also perform very well when trained with larger datasets. The CNN model for this study was trained with 27,588 data samples compared to Syarief and Setiawan (2020) who used a few data samples (200) for model training in the detection of maize diseases. The majority of the studies have employed transfer learning to train deep learning models for maize diseases detection and their scope is not focused on Tanzania (Darwish et al., 2020; Syarief and Setiawan, 2020; Chen et al., 2021; Arnaud et al., 2022; Haque et al., 2022), unlike the study where both CNN and ViT deep learning models were developed from scratch and the study area is Tanzania. Another study by Sibiya and Sumbwanyambe (2021) developed a deep learning model for early detection of maize disease using a segmentation approach, while the approach of the study for our case was classification. Furthermore, none of the studies has come up with a combined deep-learning model for the early detection of MSV and MLN diseases in maize. Additionally, when the developed deep learning models were compared, the ViT model had somewhat greater accuracy than the CNN model. According to Dosovitskiy et al. (2020), the ViT model’s key design which includes the ability to capture global dependencies through self-attention mechanisms gives it an advantage in detecting and classifying various plant diseases with higher accuracy than the CNN model. Furthermore, ViT divides the input image into patches and processes these patches as sequences, enabling the model to learn a high-resolution and systematic representation of the image data. However, when the prediction speed for both models per image is compared. CNN is 10 milliseconds faster than ViT which is 20 milliseconds per image.

## 6 Conclusion

This study has shown that early maize disease detection is possible in Tanzania, with a specific focus on the Maize Streak Virus (MSV) and Maize Lethal Necrosis (MLN). The study collected a substantial dataset comprising 26,913 field-acquired images and 675 wrong images acquired from open-access databases. The dataset’s availability as an open-source resource will facilitate further research on MSV and MLN infections. Deep learning models, namely, convolutional neural networks (CNNs) and vision transformers (ViTs), were developed to address the challenge of early disease detection. Both models were developed from scratch, with CNN demonstrating its ability to extract local image features, while ViT demonstrated proficiency in understanding the global image context. ViT achieved a validation accuracy of 93.10%, while CNN achieved a validation accuracy of 90.96%. This highlights the value of deep learning models in the early diagnosis of plant diseases in maize.

## Data Availability

The datasets presented in this study can be found in online repositories. The names of the repository/repositories and accession number(s) can be found at: https://data.mendeley.com/datasets/fkw49mz3xs/1.
